# European residential wood pellet trade and prices dataset

**DOI:** 10.1016/j.dib.2020.106254

**Published:** 2020-09-03

**Authors:** Fabian Schipfer, Lukas Kranzl, Olle Olsson, Patrick Lamers

**Affiliations:** a Technische Universität Wien E370-3, Gusshausstraße 25-29, Vienna, Austria; b Stockholm Environment Institute (SEI), Linnégatan 87D, Stockholm 115 23, Sweden; c National Renewable Energy Laboratory, 15013 Denver W Pkwy, Golden CO 80401, United States

**Keywords:** Wood pellets, Residential heating, Trade flows, Price development

## Abstract

The European market for wood pellets used in small-scale heating systems has been expanding significantly over the past decade. For an analysis of market efficiency in the Journal Energy with the title “The European wood pellets for heating market - price developments, trade and market efficiency“ wood pellet prices have been collected as well as trade flows downloaded for the trade relations between Austria, Germany, Italy and France. Only since January 2012 monthly wood pellet trade data is published by Eurostat. This, now monthly expanding data-set provides new opportunities for analysing the development of this important renewable energy commodity. Furthermore, national wood pellet prices published by national authorities and interest groups are improving in quality in the recent years. The collection and combination of these data-sets are a chance for novel econometric analysis. This paper presents valuable tools and processes to acquire and prepare this data and connects to a data and code repository for downloading the resources described in this and the related Journal Energy publication.

## Specifications Table

**Table:** 

Subject	Economics, Econometrics and Finance
Specific subject area	Economics and Econometrics
Type of data	Table Figure Macro Excel R-code
How data were acquired	Via SDMX from Eurostat as well as manual collection from several internet sites and personal communication
Data format	Raw analysed Filtered
Parameters for data collection	(1) Monthly wood pellet trade data between Germany, Austria, France and Italy from January 2012 until March 2020, monetary values and physical quantities, imports and exports. (2) Monthly wood pellet prices from Germany, Austria, France and Italy from the earliest date available (Germany since 2002, Austria since 2001, France since 2007 and Italy since 2011). Residential prices for a purchase between 5 and 8t
Description of data collection	(1) Monthly wood pellet trade data is downloaded using a R-Studio script from Eurostat via the SDMX platform. (2) Wood pellet prices are collected manually from homepages and via personal communication using a Macro-Excel tool.
Data source location	Energy Economics Group / Technische Universität Wien Vienna Austria
Data accessibility	Repository name: Github Fabian Schipfer Data identification number: JEN_2020_8 Direct URL to data: https://github.com/schipfer/Econometrics-JEN_2020
Related research article	Journal Energy Elsevier The European wood pellets for heating market - price developments, trade and market efficiency https://doi.org/10.1016/j.energy.2020.118636 Authors: Fabian Schipfe*, Lukas Kranzl*, Olle Olsson^+^, Patrick Lamers^x^ Schipfer Fabian, schipfer@eeg.tuwien.ac.at, *Gusshausstraße 25–29 Technische Universität Wien E370–3; ^+^Stockholm Environment Institute (SEI), Linnégatan 87D, 115 23 Stockholm, Sweden; ^x^National Renewable Energy Laboratory, 15,013 Denver W Pkwy, Golden, CO 80,401, United States

## Value of the Data

• Wood pellets are by traded quantity the single most important renewable energy carrier to date. Trade data and national prices are useful to understand how this commodity market is emerging, substituting energy carriers including coal, crude oil and natural gas.
• On the one hand, energy economic researchers might be interested in econometrically analysing the relationship between price and trade-developments. On the other hand, also commodity traders and financial markets experts might use this data to predict arbitrage opportunities.
• The accompanying research article in the Journal Energy uses this data to analyse the efficiency of this commodity market. Future research could focus on including additional descriptive variables to model and forecast wood pellet production, consumption and trade.
• Only since January 2012 monthly wood pellet trade data is published by Eurostat. This, now monthly expanding data-set provides new opportunities for analysing the development of this important renewable energy commodity. Furthermore, national wood pellet prices published by national authorities and interest groups are improving in quality in the recent years. The collection and combination of these data-sets are a chance for novel econometric analysis.

## 1 Data Description

### 1.1 Wood pellet trade and EUROSTAT

Harmonised statistical approaches on trade of wood pellets to provide information to the market actors have been called upon since the publication of ETA et al., [1]. Until 2009 wood pellet trade was documented in Eurostat under the trade code for “wood waste & scrap” or “sawdust”, both stating “whether or not agglomerated in logs, briquettes, pellets or similar”. Between 2009 and 2012 European statistics used a specific trade code “sawdust and wood waste scraps, agglomerated in pellets”. Sikkema et al., [2] specified the need for a double-entry bookkeeping system for intra-European trade and the documentation of extra-European imports and exports in Eurostat. In January 2012 an international pellet code was introduced by the World Customs Organisation in line with the Harmonised System nomenclature (HS 440,131). The trade code was adopted by the European Union (EU) Combined Nomenclature system (CN), thus listing wood pellet trade explicitly in national statistics of Member States (MS) and in Eurostat.

Trade flow data from [3],more specific from the International trade in goods statistics (ITGS) is used to perform the analysis in the Journal Energy paper “The European wood pellets for heating market - price developments, trade and market efficiency”. European Member States (EU MS) are obligated to report monthly import and export volumes of their goods in quantity and value. Trade streams between the EU MS (intra-EU trade) and between the MS and non-EU countries (extra-EU trade) are published online^1^ based on a harmonised approach. National statistical authorities (NSAs), mostly national statistical institutes are in charge with collecting trade data from any businesses (Provider of statistical information – PSIs) and sending them to Eurostat within the legal deadlines.

In practice, intra-EU wood pellet trade data is reported monthly from companies in EU MS which are exceeding exemption thresholds fixed on national level. For example, any Austrian business with traded monetary values (in any direction) above 750.000 € in the past or current year has to report its imported and exported values and quantities as well as additional information including used transport modes, partner countries and country of origin into the official national INTRASTAT online tool [4]**.** Exemption thresholds can vary between MS, however MS “have to ensure that at least 97% of their dispatches (intra-EU exports) by value (95% up to 2013) and 93% of their arrivals (intra-EU imports) by value are covered” (Eurostat, 2015). For successfully submitting an INTRASTAT declaration, an existing CN code (Combined Nomenclature) is required.

Since the new code for wood pellets is available, former CN codes used for this commodity are no longer listed (former subheading 440,130) and nearly all companies declare their trade electronically and companies were informed well before introduction of new codes.^2^ NSAs furthermore check the received data for plausibility, if necessary countercheck it with the declarants, estimate the statistical values to ensure comparability and estimate missing trade flows based on VAT (value-added tax) returns and foresight models including seasonality, working days etcetera.^3^ Finally, trade data is compiled by the NSAs for national publication and then forwarded to Eurostat where it is re-compiled in a harmonised approach. The Eurostat publication may differ from the national publication mainly due to better account for the common European border by use of adjusted concepts and definitions.

To avoid double counting for EU agglomerates, dispatches and arrivals cover goods which are in free circulation in the receiving MS. Imports and exports on the contrary include goods placed under customs procedure for release into free circulation in the MS of entry or after transfer to another MS. This means that a trade from country A to country B and a subsequent transfer to country C will be declared as imports of country B if put under customs control in country B. Goods in simple transit, entering and leaving a MS, with the exclusive purpose of reaching another MS or country are not recorded at all in the ITGS. However, due to customs simplification (“Single authorisation for simplified procedures” - SASP), customs can be declared in the MS of origin of a company which imports or exports goods in and from another country, which consequently leads to higher reported imports and exports in some countries than actually physically traded.

Exports and dispatches are said to be FOB type values (free on board) while CIF type values (cost, insurance, freight) are used for imports and arrivals. In simplification, this means that transportation costs originating in the sending country are included until the border of the receiving country excluding customs, excise duties or VAT. Depending on the different data collection procedures of the Member States, NSAs also estimate the statistical (at the border-) values by adding or subtracting transport costs. Therefore, NSAs also collect information about transport modes used for the respective trade.^4^ In case of any other transport mode than sea or inland waterway traders normally use the incoterms FCA (Free Carrier) and DDP (Delivery, Duty Paid) for exports/dispatches and imports/arrivals respectively.^5^ NSAs have to recalculate in order to achieve the harmonised FOB and CIF type values for all transport modes. Quantities as statistical values are collected by the NSAs rounded in full kilogram without packaging. MS are not obliged to collect this so called net mass, but have to estimate it to meet the Eurostat data requirements.

**Fig. 1:**
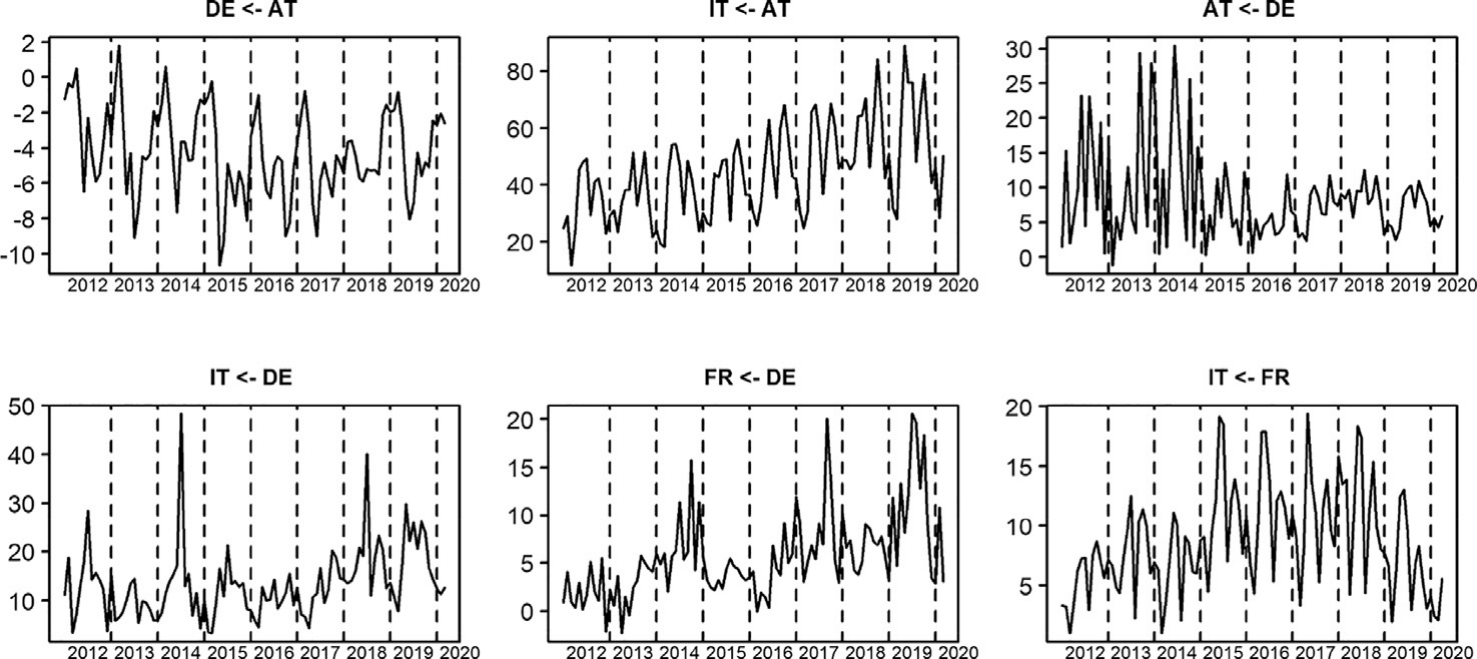
Most important trade streams between the main European wood pellets for residential heating markets. The y-axis indicates monthly net-exports in kilo tonnes. Dotted vertical lines indicate the December of each year. Source: [10].

While the main receiving country of wood pellets for heating in the EU was Italy (with 14,4 Mt over 100 months), Austria and Germany were both important wood pellets trading countries, in terms receiving pellets and sending them to the other focus countries. Imports to Italy are followed by imports to Germany (3,3 Mt), Austria (2,9 Mt) and France (1,7 Mt) over the discussed time frame. 37% of Italian imports originated from the other focus countries. France imported 21%, Austria 24% and Germany 11% from other focus countries. Italy and Austria also exhibit a seasonal pattern for total wood pellet imports but no such patterns can be found for German and French imports. For Italy and Austria import lows are around January-March while imports increase again starting with May. Net-trade flows between the focus countries are summarised in Table 1 and illustrated in Fig. 1.

**Table 1:** Net-trade of trade quantities between reporter and partner between Jan.2012 and Mar. 2020.

Partner	Reporter	Minimum	Maximum	Mean	Standard Deviation
DE	AT	−10,619,3	1799,3	−4186,2	2496,1
IT	AT	11,895,3	89,032,9	44,618,6	16,062,2
AT	DE	−2372	20,539,8	5727,2	4547,3
IT	DE	3192,3	48,233,9	13,472,2	7212,6
FR	DE	−1115,1	30,406,2	8526,4	6535,8
IT	FR	972	19,360,5	8716,7	4403,8

Even though the focus countries have been selected to present the most important EU countries where wood pellets are used for heating, we do not observe strong trade links between all of them: Only Germany and Austria indicate a strong bilateral trade i.e., in both directions (1.111 kt to Austria and 260 kt to Germany). Trade from Austria to Italy accounts for the largest single observed trade (4.455 kt) however, this trade is almost entirely uni-directional. Also, Germany and France sent a considerable share (1.334 kt) and (871 kt) of their wood pellets to Italy in the considered time frame. Trade between Germany and France is dominated by pellets sent to France (647 kt).

### 1.2 Collection of residential wood pellet prices

Residential wood pellet prices were collected from national pellets associations and national statistics for the MS of interest: Austrian residential wood pellets are based on the “Pelletpreisindex PPI06” from [11]. The Austrian wood pellet association queries prices based on private end user prices, including VAT, not packed and for an ordered amount of 6 t Furthermore, it reports a feed-in-flat-rate (“Einblaspauschale”) for direct delivery of wood pellets by a dedicated truck and pumping/blowing them into the residential pellet storage. The “Einblaspauschale” is estimated with an average of 39 € for each delivery (about 6.5€/t). German wood pellets prices are collected by “Deutsches Pelletsinstitut” (DEPV) for several regions (South Germany, North Germany and Central Germany) for different quantities delivered^6^ excluding VAT but including all costs for delivery up to 50 km [5]. The pellet price time series for 6 t delivery was further used for our analysis. Residential wood pellets are mainly sold in 15 kg bags in Italy. The “Associazione Italiana Energie Agroforestali” (AIEL) collects wood pellet prices on “retail level” without transport costs and VAT for bag and bulk purchases.^7^ For the focused time range (2012–2020) of this study pellet prices for Italy are only available every two to three months. In France official statistics publish pellet prices for 5 t deliveries up to 50 km and bag purchases (yard sale) including VAT [9]. The former values are used for this study.

To increase comparability of prices between the analysed MS we discuss only bulk delivery. For Austria the constant feed-in-flat-rate is also added to increase comparability since no time series for such flat rates can be acquired. Taxes are normally paid on pellets as delivered, i.e. total costs including transport. The changes in value added tax for wood pellets in the focus countries are illustrated in Table 2.

**Table 2:** Value added taxes for wood pellets for residential heating purposes. Source: based on [6], [7], [8], and personal communication.

Year	AT	DE	IT	FR
2012	10%	7%	10%	7%
2013	10%	7%	10%	7%
2014	10%	7%	10%	10%
2015	10%	7%	22%	10%
since 2016	13%	7%	22%	10%

**Fig. 2:**
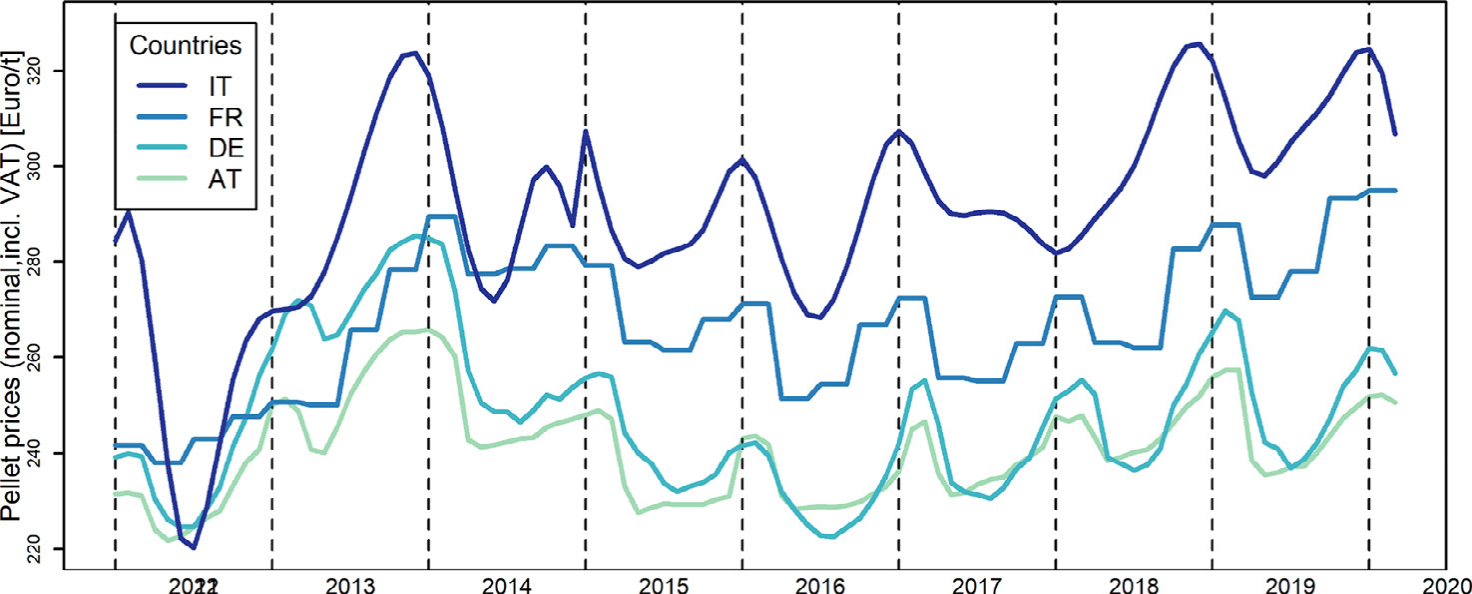
Residential wood pellet prices for the considered time period. Dotted vertical lines indicate December of each year. Source: ProPellets, (2020), [5], (Beyond20/20, 2020) and personal communication.

Wood pellet prices between 2012 and 2020, as well as price differences between main trading partners are summarised in Table 3 and illustrated in Fig. 2.

**Table 3:** Residential wood pellet prices (row 1 to 4) and price differences (row 5 to 9) for the considered time period (without VAT). Source: [11], [5], [9] and personal communication.

Set	Minimum	Maximum	Mean	Standard Deviation
AT	221,76	265,89	241	10,57
DE	222,54	285,61	247,64	15,84
IT	220,27	325,64	290,4	21,58
FR	238	294,9	267,06	15,08
AT-DE	−6,07	30,12	6,65	7,23
AT-IT	−4,38	75,24	49,4	16,87
DE-IT	−4,27	73,37	42,75	19,5
DE-FR	−21,37	46,13	19,41	15,3
FR-IT	−22,72	52,29	23,34	14,08

Lowest average wood pellet prices in the period Jan.2012-Mar.2020 can be found for Austria, followed by Germany and France while highest prices are collected for Italian consumers (see Table 3). Prices are in general higher in the winter months for all countries than during the year (Fig. 2).

## 2 Experimental design, materials, and methods

Data preparation, manipulation and subsequent econometric analysis have been performed in the statistical environment from R-Studio Version 1.3.959 running the R version 4.0.2 (2020–06–22).

In order to download the trade data points from Eurostat and automatically compile them into a panel data format, useful for econometric analysis, the Statistical Data and Metadata Exchange (SDMX) connection via the rsdmx-package [12] was used. The package can be utilised to download the data-set based on a static Uniform Resource Locator (URL)-address. The address is a combination of the Eurostat homepage, the SDMX folder, the dataset, the reporter name (Austria, Germany, France, Sweden), the traded product (wood pellets), the trade direction (import vs. export), the unit (Quantity in 100 kg vs. value in euros), and the starting period (2012). An example of a workable URL-link for monthly (M.) trade data reported by Austria (AT.) for wood pellets (440,131.) imports (1.) and physical values (QUANTITY_IN_100KG) is stated in Table 4. The downloaded data was collected into a data frame for further manipulation and evaluation.

**Table 4:** Exemplary URL-link to access wood pellet trade data via the sdmx platform from Eurostat.

http://ec.europa.eu/eurostat/SDMX/diss-web/rest/data/DS016893/M.AT..440131.1.QUANTITY_IN_100KG?startperiod=2012

**Fig. 3:**
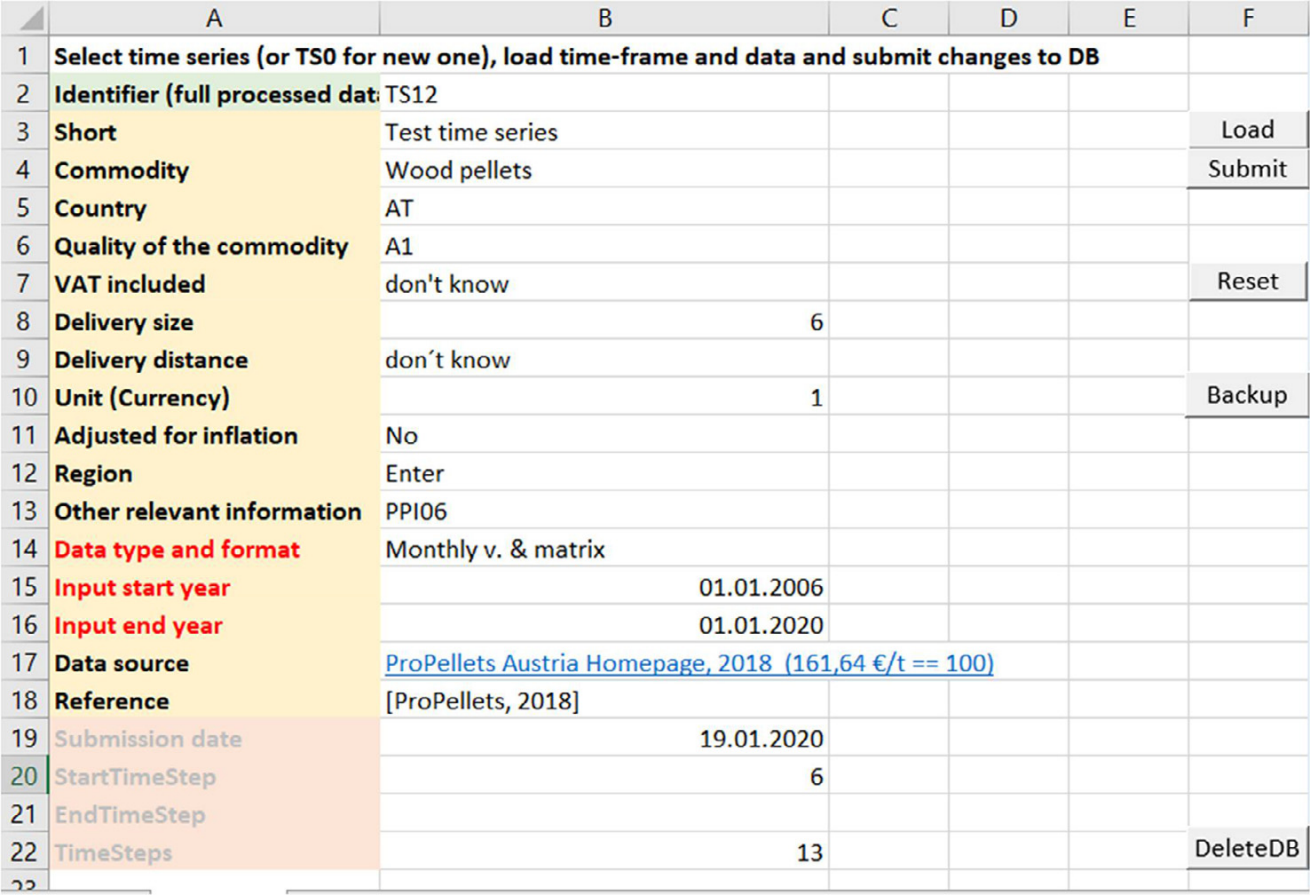
Landing page of the Price Time Series Collection Tool (PTSC-Tool) Macro Excel. Source. Own illustration.

National wood pellet prices can be found online in different formats. Data is presented on Homepages (Austria, Germany), Newsletters (Italy) and data repositories of Statistical Authorities (France, Switzerland) in the format of a column, row or data table in ascending or descending order with monthly or quarterly coverage. To decrease the susceptibility to errors of the manual data collection work and to store price data in a unified spread-sheet that is machine readable and can be directly loaded into the statistical environment of R-Studio, a Macro Excel tool was prepared. The landing page of the prices time series collection tool (PTSC-Tool) is illustrated in Fig. 3.

The submitted time series are stored in a column format starting with row #2 at January 2001 and ascending with monthly time steps. Meta-data is stored in a separate “Time series identity” spread sheet. The columns are loaded into R-Studio using the readxl-package [13]. The original time series still have to be corrected based on if they include VAT or delivery costs.

## Declaration of Competing Interest

The authors declare that they have no known competing financial interests or personal relationships which have, or could be perceived to have, influenced the work reported in this article.

## Appendix Supplementary materials

Image, application 1

## Footnotes

1 http://epp.eurostat.ec.europa.eu/newxtweb/

2 At least in Austria, personal communication 23.03.2016 Tamara Schmidt, Statistik Austria

3 To acquire and compare these foresight models for the discussed MS was out of scope of this study

4 However only transport modes for imports and exports can be downloaded on Eurostat so far. Transport mode for Intrastat-trade are not online yet.

5 Personal communication with Michael Wild, Wild / Partner LLC, Principal, 09.09.2016

6 Personal communication with Jan Schlaffke/ DEPV 13.12.2016 per e-mail and https://depv.de/de/pelletpreis accessed 15.01.2020

7 Personal communication with Laura Bau/ AIEL 02.02.2017 per e-mail and http://aielenergia.it/pubblicazioni.php accessed 15.01.2020
